# The Pharmaceutical Composition of Rocuronium Bromide May Promote Catecholamine Release From PC-12 Cells

**DOI:** 10.7759/cureus.76537

**Published:** 2024-12-28

**Authors:** Tomoaki Itaya, Shunichi Takagi, Takefumi Kamiya, Keisuke Nakazawa, Seidai Katagiri, Takahiro Suzuki

**Affiliations:** 1 Anesthesiology, Nihon University School of Medicine, Tokyo, JPN

**Keywords:** hypertensive crisis, paraganglioma, pc-12, pheochromocytoma, rocuronium

## Abstract

Background: Several cases of pheochromocytoma presenting with hypertensive crises after anesthesia induction, possibly caused by rocuronium injection, have been reported. Rocuronium has two compositions: rocuronium bromide (RB) in sodium acetate hydrate/acetic acid buffer solution (acetic acid vehicle) and RB in glycine/hydrochloric acid buffer solution (hydrochloric acid vehicle). This study assessed the effect of rocuronium composition on the release of catecholamine from PC-12 rat adrenal pheochromocytoma cells.

Methods: PC-12 cells (3x10^5^ cells) were cultured for 96 hours, then 1 mL of reagent was added for five minutes. First, the experiment used phosphate-buffered saline (PBS (-)), RB in PBS (-), an acetic acid vehicle, and RB in the acetic acid-based vehicle. Next, the experiment used an acetic acid vehicle and a hydrochloric acid vehicle. Then the experiment used an acetic acid vehicle and a mixed solution of 0.2 mL acetic acid vehicle + 0.8 mL normal saline (NS). Cell supernatants were collected and norepinephrine (NE) and dopamine (DA) concentrations were measured using high-performance liquid chromatography.

Results: Release of NE and DA was caused by acetic acid vehicle, not by RB in PBS (-). Hydrochloric acid vehicles similarly showed a significantly lower release of NE and DA than acetic vehicle acid (NE, P=0.002; DA, P=0.002). In addition, the acetic acid vehicle diluted 5-fold with NS showed a significantly lower release of NE and DA than the acetic acid vehicle alone (NE, P=0.005; DA, P=0.002).

Conclusions: The concentration of acetic acid in the buffer solution, not RB, caused the release of catecholamines from PC-12 cells.

## Introduction

The annual incidence of pheochromocytomas and paragangliomas in the general population is three to eight per million persons per year. These pathologies can cause hypertensive crises due to massive surges in catecholamines as a stress response [1]. Patients with undiagnosed pheochromocytoma could potentially undergo surgery for other reasons, and several cases of undiagnosed or diagnosed pheochromocytoma presenting with hypertensive crisis after anesthesia induction have been reported [2-5]. In such cases, rocuronium has been suspected as the cause. We reported that simultaneous measurement of plasma concentrations of rocuronium and norepinephrine (NE) by high-performance liquid chromatography in patients with paraganglioma revealed a direct correlation between the administration of rocuronium and the release of NE [2]. Several possibilities regarding the actual cause must be considered. Muscarinic receptors are located in both the atrial pacemaker and papillary muscle, and rocuronium is known to exert opposing effects on M2 muscarinic receptors [6]. In addition, rocuronium might increase NE release from human atrial tissue by inhibiting muscarinic receptors [7]. Rocuronium is also known to induce severe burning injection pain resulting in withdrawal movement, [8] which could potentially precipitate a hypertensive crisis. However, the exact mechanisms underlying hypertensive crisis after rocuronium administration remain unclear.

Rocuronium has two compositions: the original composition (e.g., Esmeron^®^ (Eslax^®^), MSD Co., Tokyo, Japan) and a modified composition (e.g., Rocuronium Bromide Intravenous Solution^®^, Maruishi Pharmaceutical Co., Osaka, Japan). The modified composition of rocuronium, using a low-acid concentration glycine/hydrochloric acid buffer solution, entered the Japanese market in 2017. Some reports have described a reduction in the frequency and severity of the withdrawal reflex after rocuronium injection using this modified composition [9,10]. However, the direct effects of rocuronium on paraganglioma or pheochromocytoma cells (such as PC-12 rat adrenal pheochromocytoma cells) have not previously been reported [11]. In addition, no reports have compared the effects of the different compositions of rocuronium on PC-12 cells. This study, therefore, investigated the direct effects of rocuronium on PC-12 cells. We hypothesized that rocuronium would increase catecholamine release from PC-12 cells, but that such effects would be reduced with the modified composition of rocuronium.

## Materials and methods

PC-12 cell line and reagents

PC-12 cells are a noradrenergic clonal line originating from rat adrenal pheochromocytoma cells [11]. PC-12 cells were obtained from RIKEN BioResource Laboratory (Ibaraki, Japan). Rocuronium bromide (RB) obtained from Selleck (S1397; Tokyo, Japan) was diluted with phosphate-buffered saline (PBS (-); Nissui Pharmaceutical Co., Tokyo, Japan). Vehicle for the original rocuronium composition (acetic acid vehicle) comprising 2 mg/mL sodium acetate hydrate (FUJIFILM Wako Pure Chemical Corporation; Osaka, Japan) and 3.3 mg/mL sodium chloride (FUJIFILM Wako Pure Chemical Corporation) was adjusted to pH 4.0 with acetic acid (FUJIFILM Wako Pure Chemical Corporation; Osaka, Japan) [12]. The vehicle for the modified rocuronium composition (hydrochloric acid vehicle), comprising 5.5 mg/mL glycine (Sigama Aldrich Japan; Tokyo, Japan) and 5 mg/mL sodium chloride, was adjusted to pH 3.0 with hydrochloric acid (FUJIFILM Wako Pure Chemical Corporation) [13]. Normal saline (NS) was obtained from Otsuka Pharmaceutical Factory (Tokyo, Japan). Concentrations of RB were set at 16 µg/mL. This was based on a case we reported in which a 41 kg patient received 30 mg of rocuronium intravenously [2], and showed a maximum plasma rocuronium concentration of 15,551 ng/mL according to Wierda simulation [14].

Cell culture

PC-12 cells were cultured in Dulbecco’s Modified Eagle Medium (Gibco, Thermo Fisher Scientific K.K.; Tokyo, Japan) supplemented with 10% v/v heat-inactivated fetal bovine serum, 10% v/v heat-inactivated horse serum, and 1% v/v penicillin/streptomycin/amphotericin B solution. Cells were cultured in a humidified atmosphere of 95% air and 5% CO_2_ at 37°C.

Assays for the release of norepinephrine and dopamine

The PC-12 cell line (3x10^5^ cells) was cultured in 10 cm dishes (FG-2090: Nippon Genetics, Tokyo, Japan) for 96 hours. Cells were washed twice in PBS (-), then 1 mL of reagent was added for five minutes at 37°C in 5% CO_2_. The first experiment used PBS (-) (n=3), 16 µg/mL RB in PBS (-) (n=3), acetic acid vehicle (n=3), and 16 µg/mL RB in acetic acid vehicle (n=3). The second experiment used an acetic acid vehicle (n=3), a mixed solution of 0.2 mL acetic acid vehicle and 0.8 mL NS (n=3), a solution of 2 mg/mL sodium acetate hydrate and 3.3 mg/mL sodium chloride without adjustment of pH using acetic acid (vehicle without acetic acid) (n=3), and hydrochloric acid vehicle (n=3). The third experiment used an acetic acid vehicle (n=6) and a hydrochloric acid vehicle (n=6). The fourth experiment used an acetic acid vehicle (n=6) and a mixed solution of 0.2 mL acetic acid vehicle + 0.8 mL NS (n=6). Cell supernatants were collected without damaging the cells, then centrifuged at 144xg for five minutes and filtered with a pore size of 0.45 μm.

High-performance liquid chromatography

NE and dopamine (DA) were measured by high-performance liquid chromatography at the Japan Institute for the Control of Aging, NIKKEN SEIL Co. (Shizuoka, Japan).

Statistical analysis

We used the Kolmogorov-Smirnov test and F-test to determine whether the data showed normal distributions. The Mann-Whitney U-test was used for nonparametric data. All statistical analyses were performed using EZR (Easy R) version 1.62 [15]. Values of P<0.05 were taken as statistically significant.

## Results

**Figure 1:**
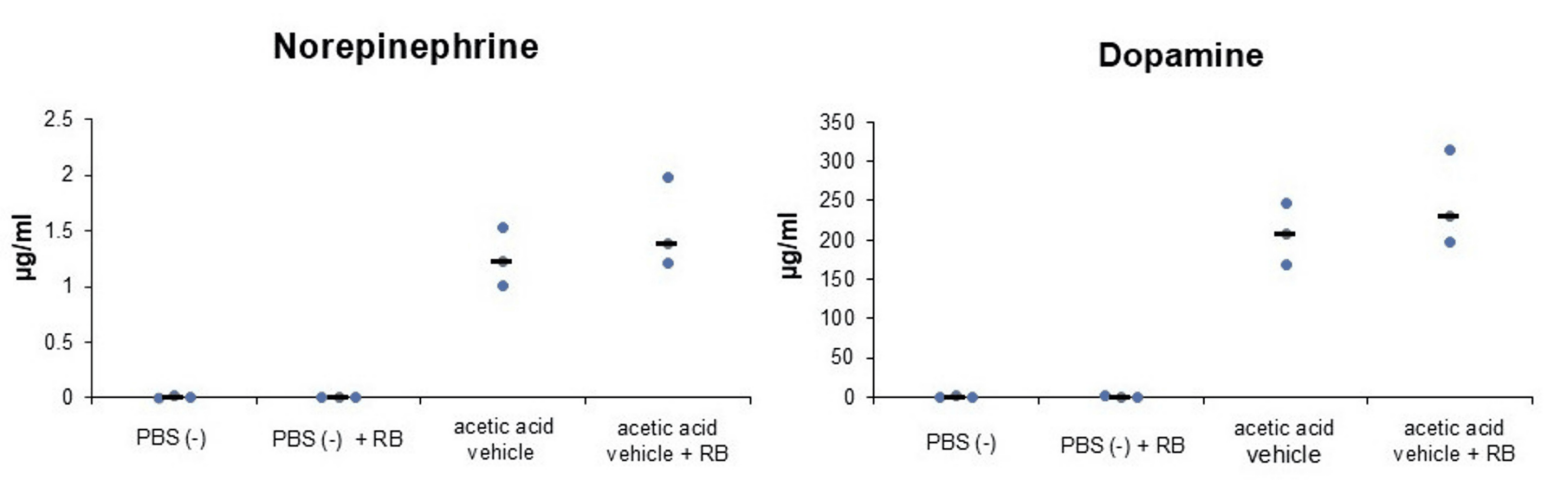
Acetic acid vehicle increased catecholamine release from PC-12 cells (median). PBS (-) (n=3) and PBS (-) + RB (n=3) did not release catecholamines from PC-12 cells. Acetic acid vehicle (n=3) and acetic acid vehicle + RB (n=3) increased the release of catecholamines from PC-12 cells. RB, rocuronium bromide; acetic acid vehicle, vehicle of the original rocuronium solution; PBS, phosphate-buffered saline

**Figure 2:**
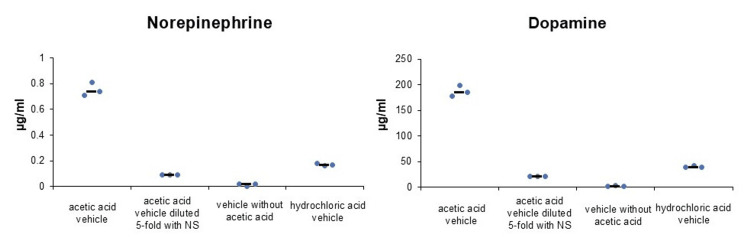
The acid concentration, rather than the lower pH, increased catecholamine release from PC-12 cells (median). Acetic acid vehicle (n=3) increased catecholamine release from PC-12 cells. Acetic acid vehicle diluted five-fold with NS (n=3), vehicle without acetic acid (n=3), and hydrochloric acid vehicle (n=3) did not increase catecholamine release from PC-12 cells. NS, normal saline; acetic acid vehicle, vehicle of the original rocuronium solution; hydrochloric acid vehicle, vehicle of the modified rocuronium solution; vehicle without acetic acid, vehicle without acetic acid of the original rocuronium solution.

**Figure 3:**
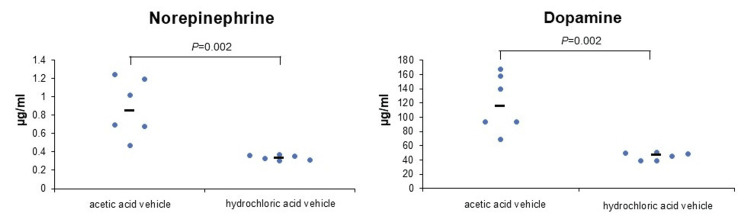
Acetic acid vehicle significantly increased catecholamine release from PC-12 cells (median). NE: P=0.002; acetic acid vehicle (n=6) vs. hydrochloric acid vehicle (n=6) using the nonparametric Mann-Whitney U-test. DA: P=0.002, acetic acid vehicle (n=6) vs. hydrochloric acid vehicle (n=6) using the nonparametric Mann-Whitney U-test. NE, norepinephrine; DA, dopamine; acetic acid vehicle, vehicle of the original rocuronium solution; hydrochloric acid vehicle, vehicle of the modified rocuronium solution

**Figure 4:**
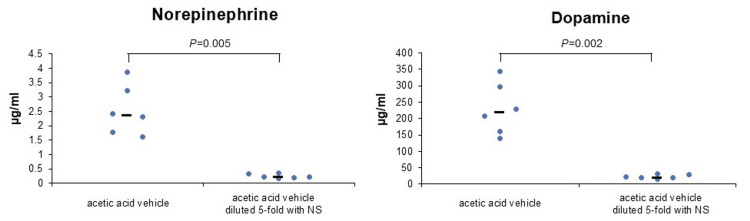
Low-acid concentration significantly decreased catecholamine release from PC-12 cells (median). NE: P=0.005, acetic acid vehicle (n=6) vs. acetic acid vehicle diluted five-fold with NS (n=6) using the nonparametric Mann-Whitney U-test. DA: P=0.002, acetic acid vehicle (n=6) vs. acetic acid vehicle diluted five-fold with NS (n=6) using the nonparametric Mann-Whitney U-test. NE, norepinephrine; DA, dopamine; NS, normal saline; acetic acid vehicle, vehicle of the original rocuronium solution

The first experiment showed that PBS (-) with or without RB resulted in almost no release of NE or DA from PC-12 cells (Figure 1). However, acetic acid vehicles released NE and DA from PC-12 cells regardless of the presence or absence of RB (Figure 1). In the second experiment, when the acetic acid vehicle was diluted five-fold with NS, the release of NE and DA from PC-12 cells was reduced approximately nine-fold (Figure 2). Vehicle without acetic acid resulted in almost no release of NE or DA from PC-12 cells, similar to PBS (-) (Figure 2). The hydrochloric acid vehicle reduced the release of NE and DA from PC-12 cells by approximately 80% compared to the acetic acid vehicle (Figure 2). In the third experiment, the hydrochloric acid vehicle achieved a significant reduction in the release of NE and DA compared to the acetic acid vehicle (NE: P=0.002, DA: P=0.002; Figure 3). In the fourth experiment, the acetic acid vehicle diluted five-fold with NS significantly reduced the release of NE and DA compared to the acetic acid vehicle (NE: P=0.005, DA: P=0.002; Figure 4).

## Discussion

To the best of our knowledge, this represents the first study to report the direct effects of rocuronium and its vehicle on PC-12 cells. In this study, RB was not seen to increase catecholamine release from PC-12 cells, counter to our expectations. In contrast, acetic acid vehicle increased catecholamine release from PC-12 cells. In this study, PBS (-) was pH 7.46, and acetic acid vehicle was pH 4.0. In addition, the acetic acid vehicle diluted five-fold with NS remained at pH 4.0 and the vehicle without acetic acid remained at pH 7.37. The acetic acid vehicle diluted five-fold with NS showed a pH similar to the pH of the acetic acid vehicle, but still reduced the release of catecholamines from PC-12 cells, meaning that low pH was clearly not the mechanism causing catecholamine release. Vehicle without acetic acid and acetic acid vehicle diluted five-fold with NS (thus containing 20% of the amount of acetic acid) both decreased the release of catecholamines from PC-12 cells. In addition, hydrochloric acid vehicle, which contained hydrochloric acid rather than acetic acid, also reduced catecholamine release from PC-12 cells.

Our results for conditions that increase catecholamine release from PC-12 cells were consistent with conditions that cause vascular pain [13]. Hydrochloric acid vehicle contains a lower concentration of hydrochloric acid buffer solution (0.03 M) than acetic acid vehicle using acetic acid (0.15 M) [13]. Jimbo et al. [13] have found that a selective TRPA1 channel inhibitor reduced the vascular pain induced by movement following the administration of an acetic acid vehicle. Wang et al. [16] reported that the degree of TRPA1 activation depends on the concentration and type of acid and demonstrated that intracellular hydrogen ions produced by weak acids such as acetic acid activated the TRPA1 channel in a concentration-dependent manner. The TRPA1 and TRPM8 channels are known as typical cold-sensing channels [17,18]. Yoshimura et al. [19] reported that PC-12 cells react to low temperatures and release transmitters by exocytosis. In addition, TRPM8 channels are expressed in PC-12 cells, and NE release is dependent on TRPM8 channels [20]. Furthermore, a high concentration of acid buffer solution could potentially have increased the release of catecholamines from PC-12 cells in a concentration-dependent manner.

In our previously reported case [2], intravenous ESLAX^®^ may have reached the paraganglioma and stimulated catecholamine release. In clinical practice, removing acetic acid is difficult, as this acid is used to adjust the pH of the original rocuronium. These findings suggest that when administering rocuronium to patients with pheochromocytoma and paraganglioma, dilution with NS may reduce the risk of hypertensive crisis.

Our study included several limitations that should be kept in mind when interpreting the results. First, PC-12 cells are a noradrenergic clonal line originating from rat adrenal pheochromocytoma cells rather than humans [11]. Second, this study was conducted in vitro, and further research is needed to determine the extent to which the results are applicable to clinical practice. However, the incidence of pheochromocytoma and paraganglioma remains very low, so a multicenter clinical study may be necessary. Third, we did not investigate the mechanism of catecholamine release from PC-12 cells.

## Conclusions

The release of NE and DA was caused by an acetic acid vehicle, not by RB in PBS (-). Hydrochloric acid vehicle similarly showed a significantly lower release of NE and DA than acetic vehicle acid. In addition, the acetic acid vehicle diluted five-fold with NS showed a significantly lower release of NE and DA than the acetic acid vehicle alone. The concentration of acid in the buffer solution, not RB, appears to be the cause of catecholamine release from PC-12 cells. Safer anesthesia may be provided to patients with pheochromocytoma or paraganglioma by diluting the rocuronium prior to administration.
